# Improved CNN for Predicting Line Heating Forming Deformation of Complex Hull Curved Plate

**DOI:** 10.3390/ma18235318

**Published:** 2025-11-25

**Authors:** Shun Wang, Haohao Jia, Yuxuan Fu, Ji Wang, Rui Li, Zhishuo Shi

**Affiliations:** 1 Naval Architecture and Ocean Engineering College, Dalian Maritime University, Dalian 116026, China; jiahaohao@dlmu.edu.cn (H.J.); yuxuan.fu@dlmu.edu.cn (Y.F.); shizhishuo@dlmu.edu.cn (Z.S.); 2 School of Naval Architecture and Ocean Engineering, Dalian University of Technology, Dalian 116024, China; wangji@dlut.edu.cn (J.W.); lirui@dlut.edu.cn (R.L.)

**Keywords:** complex curved plate, hull plate forming, intelligent prediction, strain direct boundary method, CNN

## Abstract

Shipbuilding requires numerous complex curved plates, with manual line heating forming as the primary processing method for the outer plates of the hull. To solve the problem of automation in curved plate forming, this research investigates intelligent deformation prediction for hull curved plate based on improved convolutional neural network (CNN). A numerical calculation model based on the strain direct boundary was established. The feasibility of the model was verified by comparing finite element numerical calculation results with experimental data. To achieve fast prediction of curved plate forming, a deformation prediction model for curved plate forming based on CNN was established. To further enhance the prediction accuracy of the CNN model, particle swarm optimization (PSO) and whale migration algorithm (WMA) were introduced to improve the weights of CNN, PSO-CNN model, and WMA-CNN model were established. Compared with the CNN model, the MAPE of the PSO-CNN model and the WMA-CNN model decreased by 0.31 and 1.36, respectively, for predicting the shrinkage. The MAPE decreased by 1.31 and 4.05, respectively, for predicting the deflection. This results in the WMA-CNN model effectively improving the prediction accuracy compared with the CNN model and PSO-CNN model. The WMA-CNN model is applied for the validation of the case. The relative error of the cases is all within 5%, indicating that the WMA-CNN model is applicable to the intelligent prediction of curved plate forming. This research provides reference for automation forming of hull curved plate.

## 1. Introduction

The carbon intensity indicator (CII) established by the International Maritime Organization (IMO) urgently drives the need for intelligent advancements in ship manufacturing [1]. In the process of shipbuilding, there are many complex outer plates of the hull that need to be processed. The efficiency and quality of outer plate forming directly affect the manufacturing efficiency of ships. The forming of complex curved plate is the key to achieving intelligent ship manufacturing. Line heating forming remains a primary processing method for complex curved plate [2]. Current reliance on manual operations by experienced workers in shipyards, which has disadvantages such as low processing efficiency and large fluctuations in forming quality. New technologies such as multi-point cold press have been developed in recent years [3]. However, the primary method for forming hull curved plates is line heating. Consequently, scholars have deeply investigated the line heating forming and welding of curved plate. Wei et al. [4] proposed an equivalent load model for integrated induction heating and mechanical rolling forming, validating its effectiveness through experimental forming verification of the initial complex curvature hull plate. Tong et al. [5] investigated the mechanical and fatigue properties of butt-welded high-strength steel plate, revealing significant reductions in plastic deformation capacity with increasing steel grade. Chang et al. [6] analyzed curvature effects on thermal source and stress distribution during electromagnetic induction heating, demonstrating pronounced impacts when coil width exceeds 100 mm. Shen et al. [7] developed a hybrid strategy combining mechanical rolling and laser forming for heating path planning and parameter determination in double-curved laser forming, experimentally confirming its effectiveness for complex double-curved plate with 3D shapes. Wang et al. [8] examined thermal deformation under varying power distributions during laser heating, identifying that alterations in power distributions significantly affect thermal deformation, with maximum deformation occurring under single sinusoidal power. Wang et al. [9] established a numerical model for high frequency induction heating considering skin effects and inductor motion, revealing parameter influence on plate deformation. Wei et al. [10] investigated springback control in heat-assisted incremental bending, demonstrating improved processing accuracy and efficiency. Zhou et al. [11] predicted plate-bending deformation using the thermal elastic-plastic finite element method (TEP-FEM) and the elastic finite element method for multiple line induction heating, with experimental validation. Dong et al. [12] experimentally analyzed laser parameter effects on mechanical properties and microstructure of DP980 steel plate during laser forming, identifying initial increases followed by decreases in yield strength and tensile strength with rising linear energy. Shibahara et al. [13] developed a deep reinforcement learning system integrated with line heating numerical simulation for process parameter optimization in complex curved plate forming. Zhao et al. [14] created a plastic strain database for a single heating line and developed a calculation method for required strain distribution, verifying its effectiveness in real hull plate through case studies. Current research mainly adopts three-dimensional TEP-FEM for line heating numerical simulations, with limited attention to SDB.

Currently, the level of automation in the line heating forming of curved plate is limited [15]. Sole dependence on manual experience for processing hull complex curved plate fails to meet intelligent shipbuilding requirements. Therefore, extensive research on deformation prediction has been conducted by many scholars. Lee et al. [16] examined multiple line heating effects on steel plate thermal deformation, deriving theoretical equations for predicting transverse shrinkage and angular deformation through experimental and numerical analyses. Hai et al. [17] developed an artificial neural network (ANN) for induction heating position prediction, validating its efficiency and accuracy against finite element method. Song et al. [18] established an ANN model to predict curved plate forming time by inputting hull plate geometric parameters, demonstrating higher accuracy compared to linear regression and preset equations. Li et al. [19] proposed an improved salp swarm algorithm (ISSA) integrated with extreme learning machine (ELM) for line heating deformation prediction. They verified the accuracy of the ISSA-ELM model through comparative analyses of the prediction performance of different optimization algorithms on line heating forming. Yang et al. [20] proposed a Faster region-based convolutional neural network (Faster R-CNN) model for predicting heating line locations based on the shape of the target surface. They validated the model on curved surfaces with arbitrary curvatures, demonstrating its effectiveness in locating heating lines during the line heating forming. Zhang et al. [21] developed a planning optimization method for curved plate line heating path based on multi-objective analysis and PSO. This algorithm generates heating lines according to the shape of the target curved plate, thereby minimizing repeated heating and improving efficiency. Truong et al. [22] employed a novel convolutional neural network trained on deformation distribution of desired steel plate shapes to predict induction heating lines. The model predicts sensor posture, shape, and velocity based on deformation distribution of target steel plate. Spathopoulos et al. [23] proposed a Bayesian regularized neural network-based method for springback prediction in curved plate forming, analyzing the influence of forming tool geometry, contact and friction, and sheet thickness on springback. Results from curved plate springback prediction validated the accuracy of the ANN model. Kim et al. [24] introduced a convolutional neural network-based classification method for ship-curved surfaces. This deep learning technique enables high-precision automatic classification of hull plate, thereby effectively guiding line heating forming process selection. In the existing research, there is a lack of deformation prediction models that can quickly predict curved plate forming.

To research the deformation intelligent prediction of line heating of curved plate based on improved CNN, a finite element simulation model of line heating of curved plate based on strain direct boundary method (SDB) is established in this paper. The feasibility of the model was verified by comparing finite element numerical calculation results with experimental data. To achieve fast prediction of curved plate forming, a deformation prediction model for curved plate forming based on CNN was established. The relative error between the numerical calculation values and the CNN model prediction values is analyzed. To further enhance the prediction accuracy of the CNN model, particle swarm optimization and whale migration algorithm were introduced to improve the weights of CNN; PSO-CNN model and WMA-CNN model were established. The relative error and evaluation indices of CNN model, PSO-CNN model, and WMA-CNN model are analyzed. To verify the prediction accuracy of the WMA-CNN model, the model is applied for the calculation cases validation to determine whether the relative error is within the engineering acceptable range.

## 2. Numerical Calculation of Line Heating Forming Based on SDB

To solve the problem of TEP-FEM analysis requiring a significant amount of time, a finite element simulation model of line heating of curved plate based on SDB is established and verified through experiments.

### 2.1. Line Heating Forming Mechanism of Curved Plate

**Figure 1:**
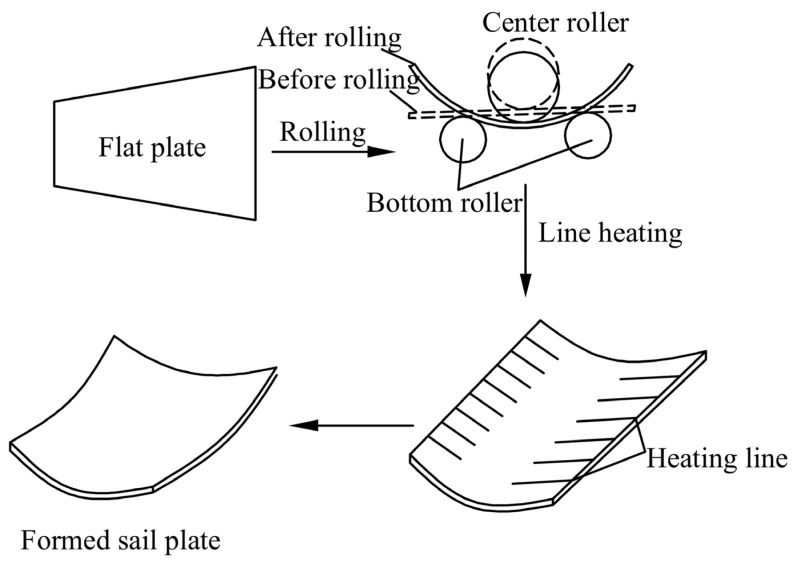
Processing of double-curved plate.

**Figure 2:**
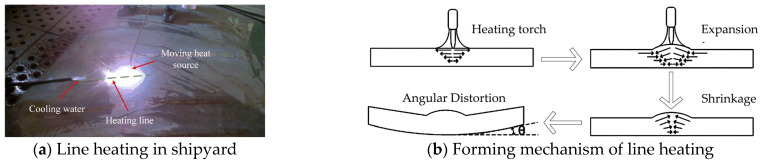
Forming process of curved plate.

Its forming method is illustrated in Figure 1. The line heating forming process is shown in Figure 2. The heat source moves uniformly along the heating line, causing rapid temperature rise in the heat affected zone. Thermally expanded metal in this zone is constrained by surrounding material. Subsequently, cooling water is introduced to cool the heating area. The temperature of curved plate began to decline sharply. During the cooling process, the generated shrinkage exceeds the expansion generated during the heating process. Therefore, irreversible plastic shrinkage is generated.

### 2.2. Loading Approach of SDB

**Figure 3:**
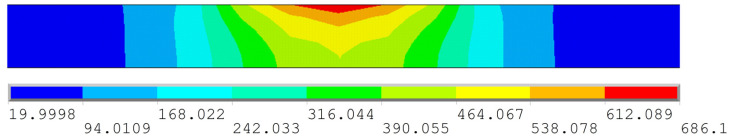
Cross section of temperature field.

The SDB applies different temperatures to the top and bottom of the model to realize the temperature gradient, which forms a non-uniform temperature field leading to deformation. The cross section of the temperature field input into the mesh by the SDB is shown in Figure 3. The temperature varies significantly along the thickness and length directions of the plate. The temperature of the upper plate surface is the highest. The temperature gradually decreases along the thickness direction. The closer to the heating line area, the higher the temperature. The further away from the heating line area, the lower the temperature. The temperature in some areas far away from the heating line has decreased to the same level as the ambient temperature.

**Figure 4:**
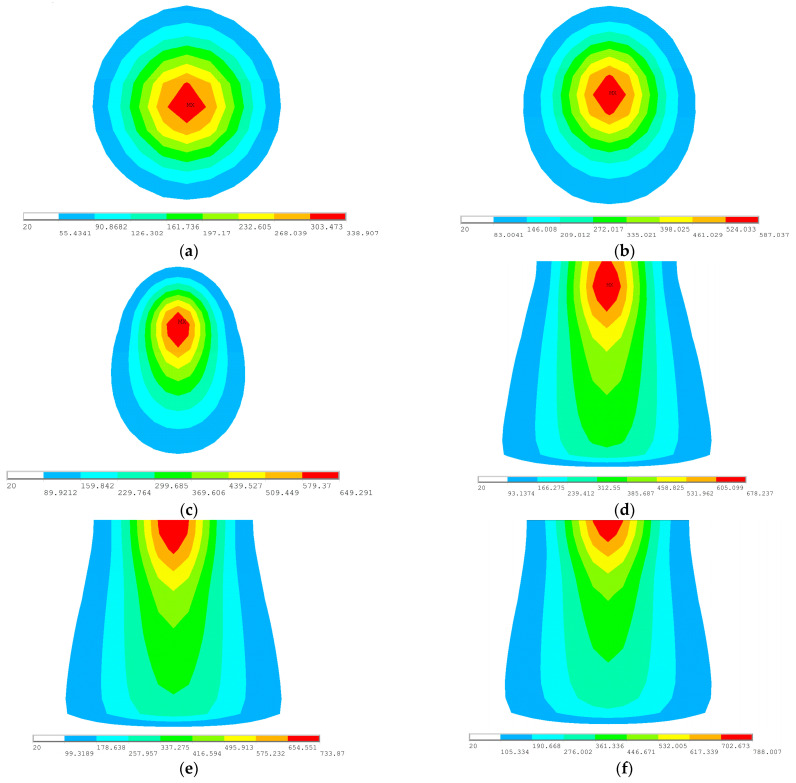
Distribution of maximum temperature along the heating line: (**a**) 5 s; (**b**) 20 s; (**c**) 42 s; (**d**) 128 s; (**e**) 132 s; (**f**) 135 s.

The initial temperature boundary input for the SDB is represented according to the Gaussian moving heat source. The distribution of maximum temperature at different times is shown in Figure 4. The period from 5 to 42 s is the initial area, 42 to 128 s is the stable area, and 128 to 135 s is the final area. During the forming process, the stable area shows the longest duration. The abrupt temperature changes occur at the initial area and the finial area of the heating line. To achieve uniform heating, the maximum temperature during the stable area is adopted as the centric temperature of the top temperature *T_t_*.

**Figure 5:**
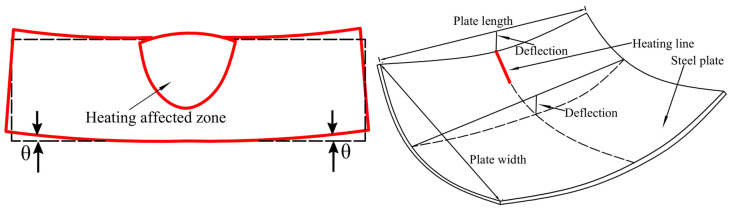
Deformation of plate based on SDB.

Along with the thickness direction, the finite element model is stratified into five layers. Table 1 presents the longitudinal and transverse temperature field calculated by TEP-FEM. The curved plate line heating forming is simulated through inputting the calculated temperatures into the model, as shown in Figure 5.

The temperature coefficient *K* is given in Equation (1):

(1) $$K_{i,j}=\frac{T_{i,j}}{T_{t}}(i=1,\ldots n.\;j=1,\ldots m)$$

where $T_{t}$ represents the centric position temperature. $T_{i,j}$ represents the temperatures of other positions. *i* and *j* stand for the row and column numbers.

### 2.3. Feasibility Verification of Numerical Calculation of SDB

A finite element simulation model of line heating of curved plate was established based on the SDB. Table 2 lists the plate parameters of finite element model. After heating, water cooling is adopted for the upper surface of the plate as the cooling method.

**Figure 6:**
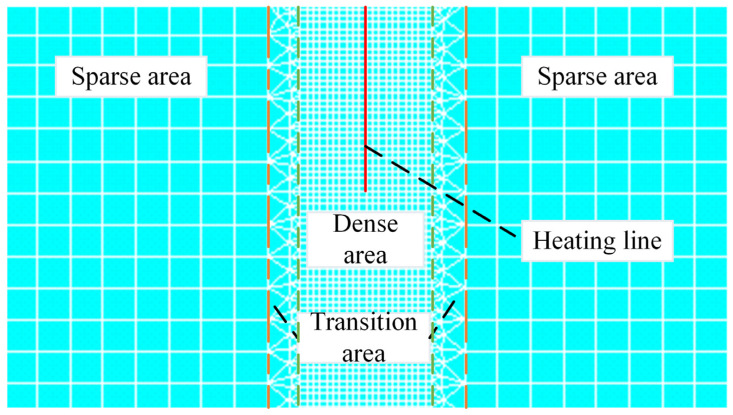
The heating line position and mesh division of the numerical calculation model.

Mesh division for the finite element model is shown in Figure 6. The area where the heating line is and the temperature gradient changes greatly is divided into the dense area. For the area far away from the heating line, the temperature gradient changes little and is divided into sparse areas. The transition area is located between the sparse area and the dense area.

The quantity of nodes in the model is determined by the mesh size, which has a major impact on the numerical results. Therefore, mesh independence verification is required. For the transition area, the mesh size is set to be twice as large as that of the dense area. For the sparse area, the mesh size is set to be five times as large as that of the dense area. Different schemes for mesh size in Table 3 are designed.

**Figure 7:**
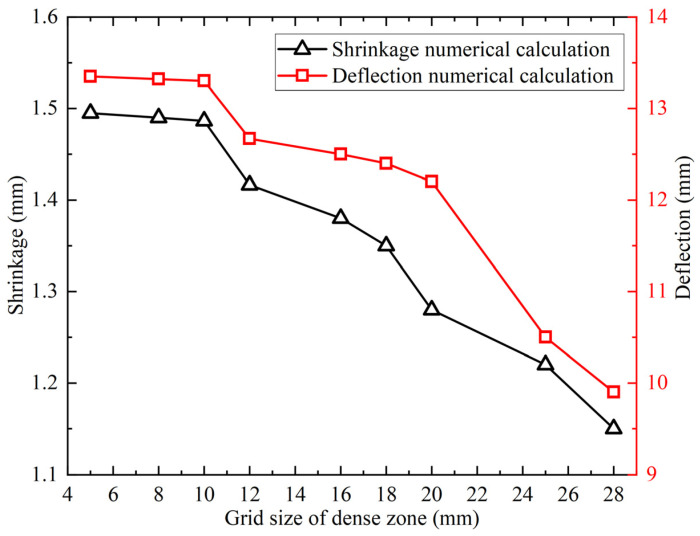
Deformation for different mesh schemes [25].

Figure 7 presents the calculated shrinkage and deflection results from the different mesh schemes. When the mesh size in the dense area exceeds 10 mm, the gradually decrease in shrinkage and deflection. When the mesh size in the dense area is smaller than 10 mm, the deflection and shrinkage of different mesh schemes are nearly identical. The relative error between the numerical results of each scheme is within 5%. The model adopts mesh sizes of 10 mm, 20 mm, and 50 mm in the dense, transition, and sparse areas.

(1) Comparison of numerical results from TEP-FEM with experimental data

Table 4 presents experimental and numerical calculation results and errors of shrinkage. The experimental shrinkage does not differ much from those of the numerical calculation results. The maximum absolute error does not exceed 0.05 mm. The final deflection of numerical calculation is 11.96 mm. The final deflection of experimental result is 11.60 mm. The absolute error is 0.36 mm. The feasibility of numerical calculation of the simulation model by the TEP-FEM was verified.

(2) Temperature results analysis for TEP-FEM and SDB

**Figure 8:**
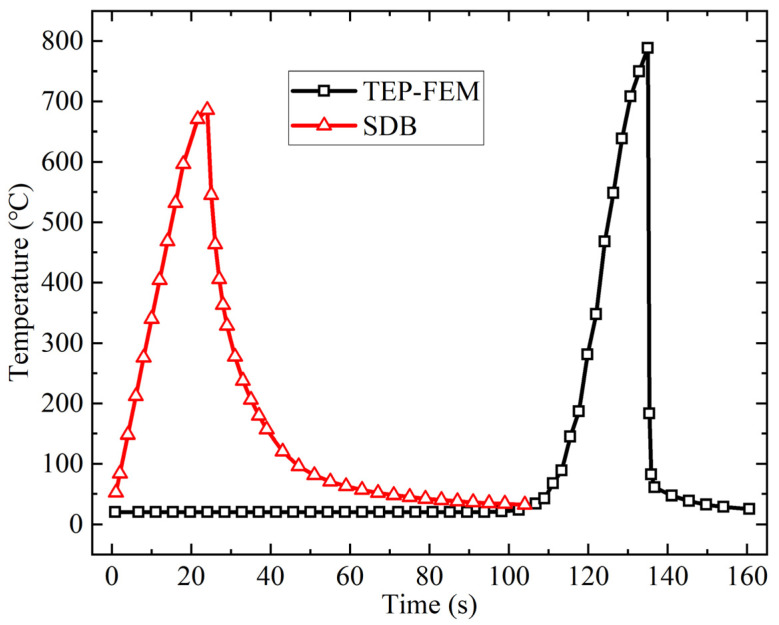
Temperature comparison of TEP-FEM with SDB [25].

Figure 8 illustrates the change in the temperature of the coincidence point between the upper plate edge and the heating line with the time, calculated by both the TEP-FEM and the SDB. For the TEP-FEM, in the initial stage of heating, the moving heat source model is far away from the upper plate edge. The midpoint of the plate length is not affected by the heat source. After 100 s, as the heat source approached the end point, the plate surface temperature gradually increased. Heating ceased at 135 s, while tracking cooling water is applied to the plate edge, causing the surface temperature to decrease to ambient temperature. In contrast, the SDB applied the temperature load to the heat affected zone, leading the plate surface temperature to reach its maximum after 24 s. Then, the plate surface temperature decreased to ambient temperature under cooling water. Figure 8 reveals the change in the temperature with time of the two methods is similar, with closely aligned maximum temperatures.

(3) Deformation results analysis for TEP-FEM and SDB

**Figure 9:**
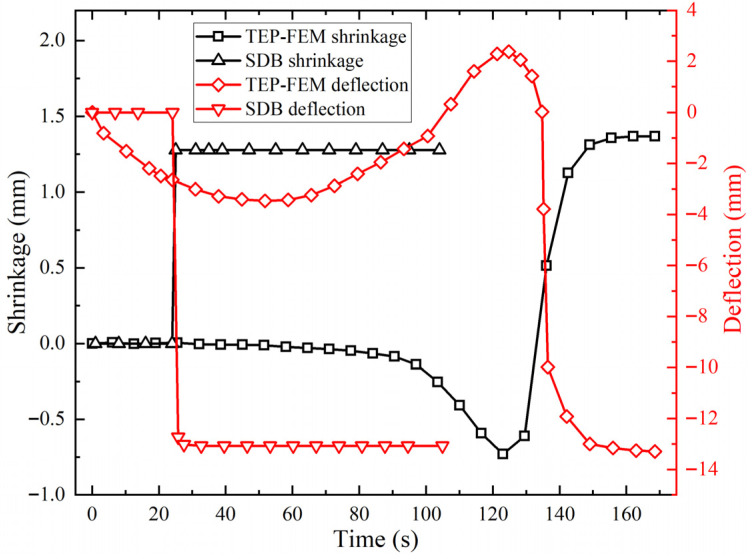
Deformation comparison of TEP-FEM with SDB.

Figure 9 shows deformation law with time for both methods. For the SDB, during 0–24 s, direct temperature loading causes large thermal expansion deformation in the curved plate. Therefore, the birth and death element method were used. Before 24 s, the elements in the heat-affected zone are set to “death”. When plate surface temperature reaches its maximum, the elements in the heat affected zone are set to “live”, and water cooling is subsequently implemented. Consequently, deformation remains zero within the first 24 s. After 24 s, units are reactivated. A rapid temperature drop induced by water cooling causes de-formation in the curved plate. The shrinkage values calculated by the TEP-FEM and SDB are 1.37 mm and 1.28 mm with a 0.09 mm absolute error. Deflection values are 13.26 mm and 13.07 mm with a 0.19 mm absolute error. The calculation errors of both methods are within acceptable limits. The feasibility of numerical calculation of the simulation model based on the SDB was verified.

## 3. Prediction of Deformation of Line Heating of Curved Plate Based on CNN

Numerous factors influence curved plate line heating forming, including both geometric parameters and forming parameters of the curved plate. The cost of obtaining deformation data of the curved plate through experiments is excessive. The calculation of a large number of samples using numerical calculation methods takes a long time. To achieve rapid prediction of curved plate forming, a deformation prediction model based on CNN is established.

### 3.1. Sampling and Processing of Samples

**Figure 10:**
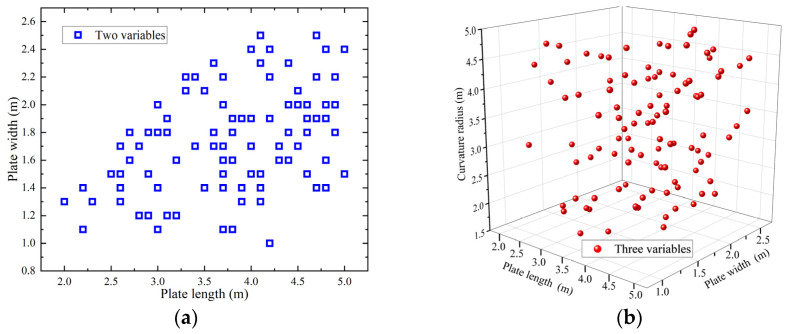
Distribution of samples by Latin hypercube sampling: (**a**) two variables; (**b**) three variables.

Before constructing the prediction model, sample points are selected within the design space. Latin hypercube sampling is adopted to ensure the sample data are uniformly distributed in all variables [26]. Latin hypercube sampling is a hierarchical Monte Carlo sampling method. This stratified Monte Carlo method divides the parameter space into multiple small areas. It conducts uniform sampling within each area and it can better cover the entire parameter space. The representativeness and reliability of sampling have been enhanced. Using plate length (2–5 m) and width (1–2.5 m) as variables, the sample distribution is shown in Figure 10a. Then, curvature radius constraints (1–5 m) are added; the sample distribution is shown in Figure 10b. Based on three variables, plate thickness (0.008–0.028 m) and heating line length (0.2–0.4 m) constraints are added. Latin hypercube sampling generated 102 numerical calculation schemes, as illustrated in Table 5.

Before the CNN-based deformation prediction model is established for curved plate, sample data normalization is essential. Normalization eliminates the influence of dimensions by mapping raw data to a 0–1 range. Prevent certain features from having an excessive impact on the model. Normalization can also enhance model performance, making it more convenient to compare the relationships between features. Applying normalization to Table 5 data transforms $X_{i}(k)$ into $X_{j}(k)$. The normalization equation is shown in Equation (2).

(2) $$X_{j}(k)=\frac{X_{i}(k)-\min(X_{i}(k))}{\max(X_{i}(k))-\min(X_{i}(k))}$$

where $X_{i}(k)$ denotes original data; $X_{j}(k)$ represents normalized data; $\max(X_{i}(k))$ is the maximum value in the original data; $\min(X_{i}(k))$ is the minimum value in the original data. The normalized results are presented in Table 6.

### 3.2. Intelligent Prediction Model for Deformation of Line Heating of Curved Plate Based on CNN

**Figure 11:**
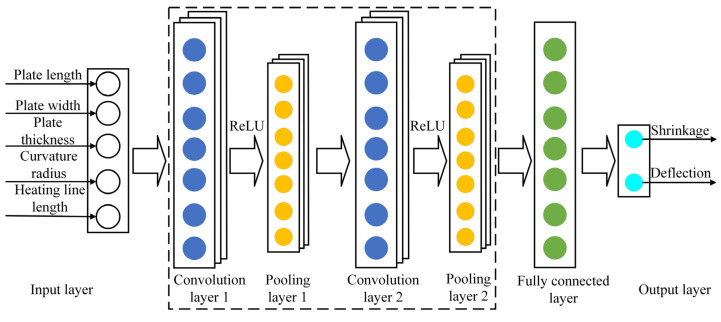
Flow chart of CNN.

The CNN model contains input layer, convolutional layer, pooling layer, fully connected layer, and output layer, as depicted in Figure 11. Feature extraction is achieved through multilayer convolution of forward propagation. The input layer includes the prediction data. Convolution kernel in convolutional layer contains weight coefficient. The pooling layer does not contain weight coefficient. These two layers constitute the core components for data processing and learning. The convolutional layer performs convolution by computing input with convolution kernel. The convolution kernel is essentially a numeric matrix that expands data dimensions to facilitate subsequent feature extraction. The pooling layer reduces data dimensionality by methods like max pooling and mean pooling. Thereby, it decreases network complexity and parameters to prevent data overfitting while simultaneously enhancing model fault tolerance. The fully connected layer processes feature data extracted by the convolutional layer and the pooling layer and achieves prediction output through the output layer.

The convolution calculation process is shown in Equation (3).

(3) $$h_{j}^{l+1}=\sigma \left(\sum_{i=1}^{F}h_{i}^{l}*w_{i\;j}^{l+1}+b_{j}^{l+1}\right)$$

where * represents the convolution operation in CNN; $h_{i}^{l}$ represents the feature map at layer *l*; $h_{j}^{l+1}$ represents the feature map at layer *l* + 1; $w_{i\;j}^{l+1}$ represents the convolutional weight at layer *l* + 1; $b_{j}^{l+1}$ represents the bias at layer *l* + 1; σ represents the activation function.

**Figure 12:**
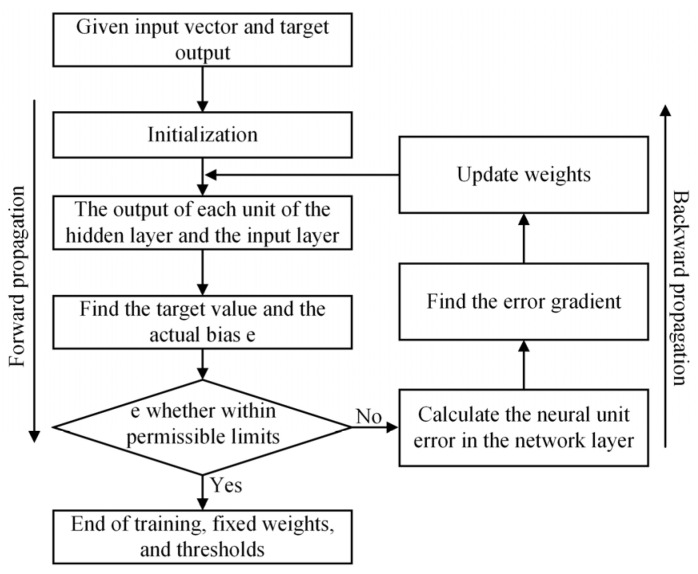
Training process of CNN.

The training of CNN is an iterative optimization process. It aims at progressively reducing deviation between model outputs and expected targets by continuously adjusting network parameters [27]. The training process of CNN is shown in Figure 12. The training process of CNN is divided into two stages: forward propagation and backward propagation. Forward propagation is the stage in which data spread from a lower level to a higher level. Backward propagation is the process of propagating errors from a higher level to a lower level when the output deviates from expectations. During forward propagation, input data undergo convolution and pooling operations for hierarchical feature extraction and ultimately produce the output result. When the outputs deviate from the expected target, the backward propagation is entered. In this stage, the network uses the error backward propagation algorithm to trace error from the output layer back to the input layer. Based on these error feedbacks, network parameters are adjusted to progressively align model outputs with expected values.

**Figure 13:**
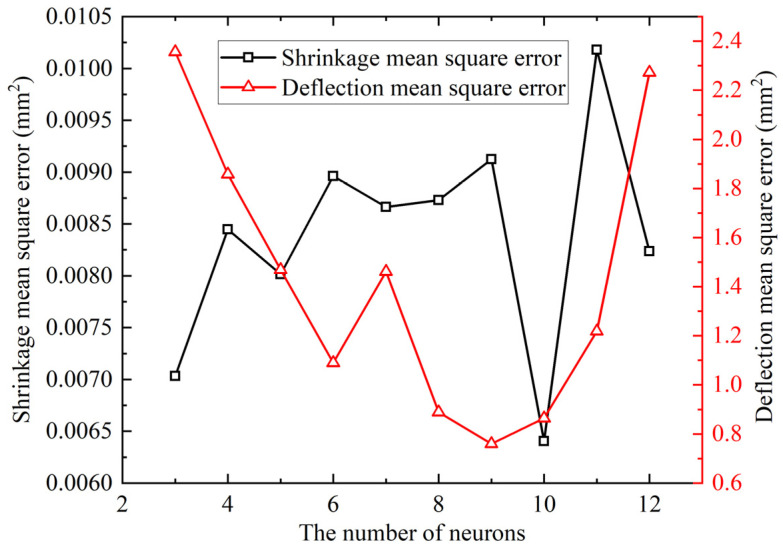
Mean square error for different numbers of neurons in the fully connected layer.

Based on the CNN, a deformation prediction model of line heating of curved plate is established. A total of 80% of samples from Table 5 formed the training set, and 20% formed the test set. Input parameters contain the following five variables: plate length, plate width, plate thickness, curvature radius, and heating line length. Prediction targets are shrinkage at 50 mm left of the heating line and deflection at the plate edge. Due to differing prediction complexities, the shrinkage prediction model and deflection prediction model are established. The input contains five feature values; the output is one value, and the number of convolutional layers is set to two. Mean squared error (MSE) of the training set under different neuron counts in fully connected layer is shown in Figure 13. Minimal MSE occurred at 10 neurons for shrinkage prediction and 9 for deflection prediction. Therefore, the fully connected layers are set to 10 and 9 neurons, respectively. The MSE equation is shown in Equation (4):

(4) $$MSE=\frac{1}{m}{\sum_{i=1}^{m}\left(y_{i}-{\overset{⌢}{y}}_{i}\right)}^{2}$$

where $y_{i}$ represents numerical simulation results; ${\overset{⌢}{y}}_{i}$ represents predicted values; m represents the number of samples in the training set.

**Figure 14:**
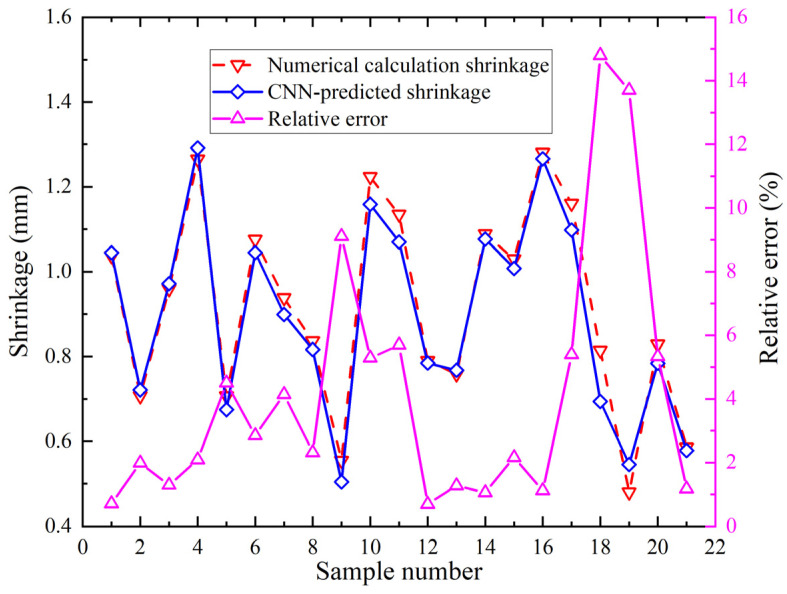
Shrinkage comparison of numerical calculation and CNN.

The CNN model utilizes geometric parameters and a forming parameter. Geometric parameters include plate length, plate width, plate thickness, and curvature radius. The forming parameter is heating line length. The prediction targets are plate edge deflection and shrinkage 50 mm left of heating line end. Therefore, the shrinkage prediction model contains five input features. Its output is one value. It contains two convolutional layers. Training iterations are 8000. The fully connected layer has ten neurons. The activation function is “ReLU”. The comparison between the numerical calculation shrinkage values and CNN predicted shrinkage values and the relative errors for twenty-one group samples are shown in Figure 14. The minimum relative error occurs in group 12 at 0.70%. The maximum relative error occurs in group 18 at 14.80%.

**Figure 15:**
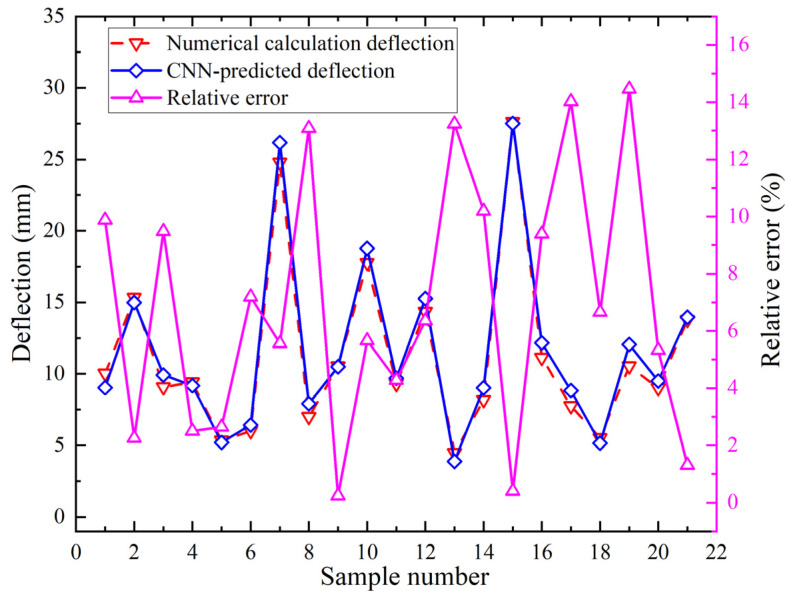
Deflection comparison of numerical calculation and CNN.

The deflection prediction model employs five input features with single value output, two convolutional layers, 8000 iterations, and nine fully connected neurons. The activation function is “ReLU”. The comparison between the numerical calculation deflection values and CNN predicted deflection values and the relative errors for twenty-one group samples are shown in Figure 15. Relative errors between predicted deflection values and numerical calculation deflection values are shown in Figure 15. The minimum relative error occurs in group 9 at 0.24%. The maximum relative error occurs in group 19 at 14.46%.

**Figure 16:**
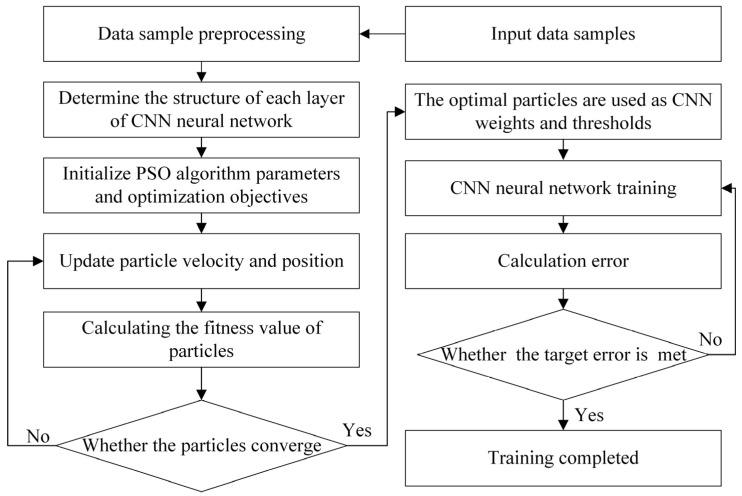
Training process of PSO-CNN.

Particle swarm optimization fundamentally leverages information sharing among individuals within a swarm. This drives the group’s movement from disorder to ordered evolution in the solution space to obtain optimal solutions [28]. The PSO algorithm simulates the predatory behavior of bird flocks. Flocks of birds are searching for the position of richest food in a forest. Each bird searches in the directions it has judgment of and records its personal position with richest food. Simultaneously, the bird flocks share all discovered food information to identify the current position with richest food. Each bird adjusts search directions based on its own memory and best position shared by the bird flocks. After some time, the bird flocks can then find the position with the richest food in the forest (global optimum). The PSO-CNN model training workflow is shown in Figure 16. The number of particles in the population of the PSO-CNN model is set to 20. The inertia factor is set to 0.5. Both the self-cognitive learning factor and the social cognitive learning factor are set to 1.

*j* Represents the particle search space dimensionality and *n* represents particle count. Each particle in the PSO algorithm represents a solution. The position of particle *i* $\left(i=1,2,\ldots n\right)$ is denoted as $x_{i}=(x_{i,1},x_{i,2},\ldots x_{i,j})$. The velocity is denoted as $v_{i}=(v_{i,1},v_{i,2},\ldots v_{i,j})$. Particles are updated through their individual best $p_{best}$ and global best $g_{best}$. When satisfying the stop condition, the optimal solution is output. The particle update equations are shown in Equations (5) and (6):

(5) $$v_{i\;j}(t+1)=wv_{i\;j}(t)+c_{1}r_{1}(p_{best\;i\;j}(t)-x_{i\;j}(t))+c_{2}r_{2}(g_{best\;i\;j}(t)-x_{i\;j}(t))$$

(6) $$x_{i\;j}(t+1)=x_{i\;j}(t)+v_{i\;j}(t+1)$$

where w is the inertia weight; $c_{1}$ and $c_{2}$ are the trade-off parameters that control between $p_{best}$ and $g_{best}$; $r_{1}$ and $r_{2}$ are random numbers regenerated per iteration within the range 0 to 1; $p_{best\;i\;j}(t)$ represents the current optimal position of the *i*-th particle in the *t*-th generation and the *j*-th dimension; $g_{best\;i\;j}(t)$ represents the global optimal position of the *i*-th particle in the *t*-th generation and the *j*-th dimension; $x_{i\;j}(t)$ represents the position of the *i*-th particle in the *t*-th generation and the *j*-th dimension; $v_{i\;j}(t+1)$ represents the velocity of the *i*-th particle in the *t*-th generation and the *j*-th dimension.

**Figure 17:**
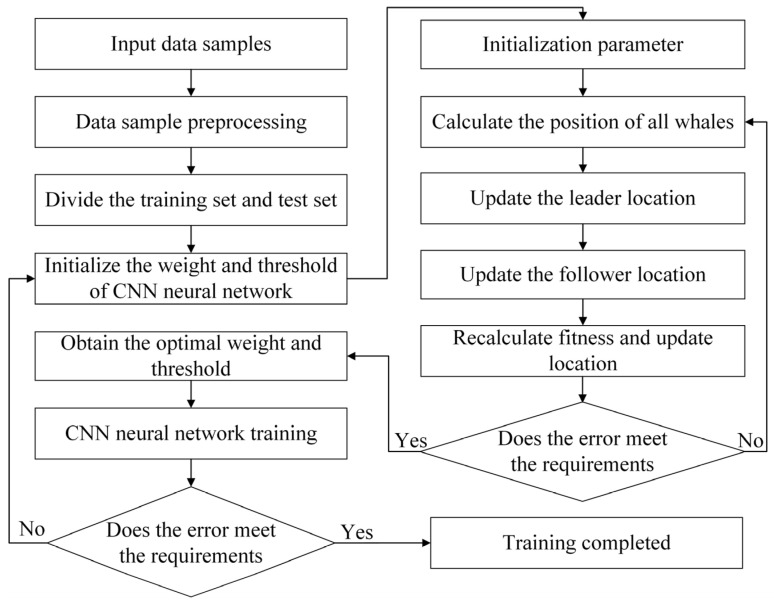
Training process of WMA-CNN.

The whale migration optimization algorithm is an optimization algorithm based on the migration behavior of whale groups [29]. Its core concept simulates whale migration behavior to achieve effective exploration and exploitation within search space. WMA algorithm, by integrating leader and follower dynamics and adaptive migration strategies, balances the relationship between exploration and exploitation, thereby enhancing capabilities to avoid local optima and converge efficiently. The WMA-CNN model training workflow is illustrated in Figure 17.

The WMA algorithm requires randomly generated set of whale locations in space. Assuming the whale position is $W=(w_{1},w_{2},\cdots ,w_{D})$, where *D* denotes the problem dimensionality. The initial position of the whale can be generated in Equation (7):

(7) $$W_{i}=L+\mathrm{rand}(1,D)⊙(U-L),\;i=1,2,\cdots ,N_{pop}$$

where $L=(L_{1},L2,\cdots ,L_{D})$ denotes the lower bound vector of search space; $U=(U_{1},U2,\cdots ,U_{D})$ is the upper bound vector; rand(1,D) is a D-dimensional random vector with elements within the range 0 to 1; ⊙ represents the Hadamard product; $N_{pop}$ is the whale group size.

Within each generation, whales are ranked by fitness values, with the highest fitness individual designated as leader $W_{Best}$ and others as followers $W_{i}$. The position of the followers is updated along with the position of the leader.

Equation (8) represents the position of the leader:

(8) $$W_{j}^{new}=W_{j}+\mathrm{rand}(1,D)⊙L+\mathrm{rand}(1,D)⊙(U-L)$$

Equation (9) represents the position of followers:

(9) $$W_{i}^{new}=W_{Mean}+\mathrm{rand}(1,D)⊙(W_{i-1}-W_{i})+\mathrm{rand}(1,D)⊙(W_{Best}-W_{Mean})$$

(10) $$W_{Mean}=\frac{1}{N_{L}}\sum_{j=1}^{N_{L}}W_{j}$$

In Equations (9) and (10), $W_{Mean}$ represents the average of the current whale positions of all leaders, $W_{i-1}$ represents the position of the previous whale of the follower whales $W_{i}$. $N_{L}$ represents the number of leader whales.

## 4. Results and Analysis of Curved Plate Forming Prediction Model

To evaluate the prediction accuracy of the CNN model, PSO-CNN model, and WMA-CNN model, the evaluation indices of these models were compared and analyzed. To verify the prediction accuracy of the WMA-CNN model, the WMA-CNN model is applied for the calculation cases validation to determine whether the relative error is within the engineering acceptable range.

### 4.1. Analysis of the Results of the Prediction Model

(1) Accuracy analysis of shrinkage prediction

**Figure 18:**
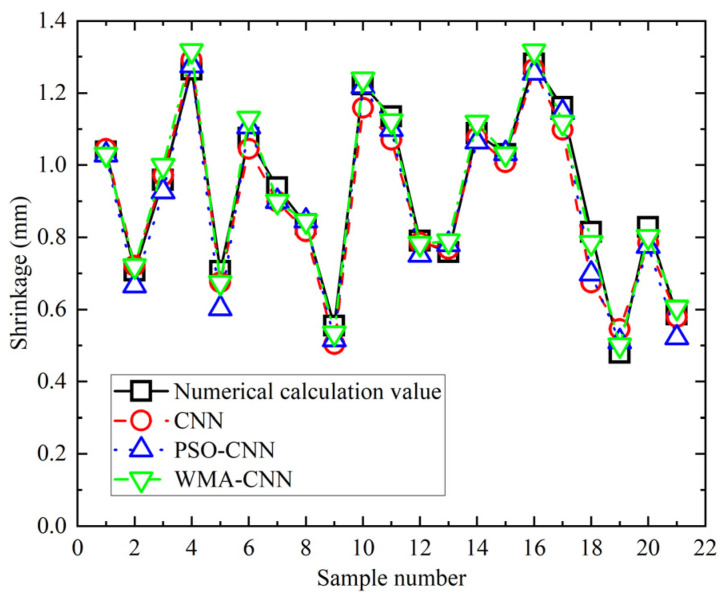
Shrinkage comparison of numerical values and predicted values.

**Figure 19:**
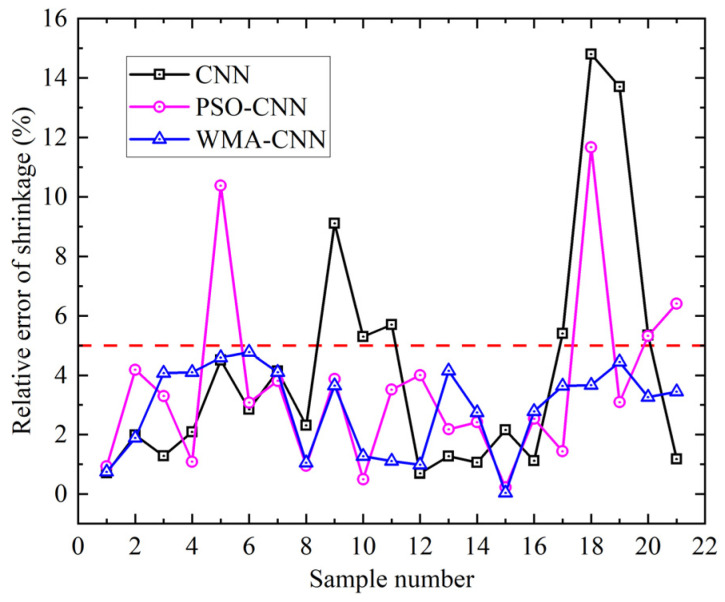
Relative error comparison of shrinkage.

Comparison of the predicted and numerical calculation values of twenty-one sets of shrinkage samples for the PSO-CNN model, the WMA-CNN model, and the CNN model is shown in Figure 18. Significant deviations are observed in CNN predictions for samples 10, 11, 17, 18, and 19. In PSO-CNN, significant deviations are shown in samples 5, 18, and 21. In WMA-CNN, marked deviations are demonstrated in samples 7 and 17. Predictions from the WMA-CNN model are closer to numerically calculated shrinkage values. The relative error of the predicted and numerically calculated values of 21 sets of shrinkage samples for the PSO-CNN model, the WMA-CNN model, and the CNN model are shown in Figure 19. The maximum relative error is observed at 14.80% for CNN, reduced to 11.67% for PSO-CNN, and further decreased to 4.77% for WMA-CNN. CNN predictions exceed 5% relative error in 7 samples, PSO-CNN in 4 samples, while all WMA-CNN values remain below 5%. The results show that the WMA-CNN model outperforms the CNN model and PSO-CNN model in terms of the shrinkage prediction accuracy.

(2) Accuracy analysis of deflection prediction

**Figure 20:**
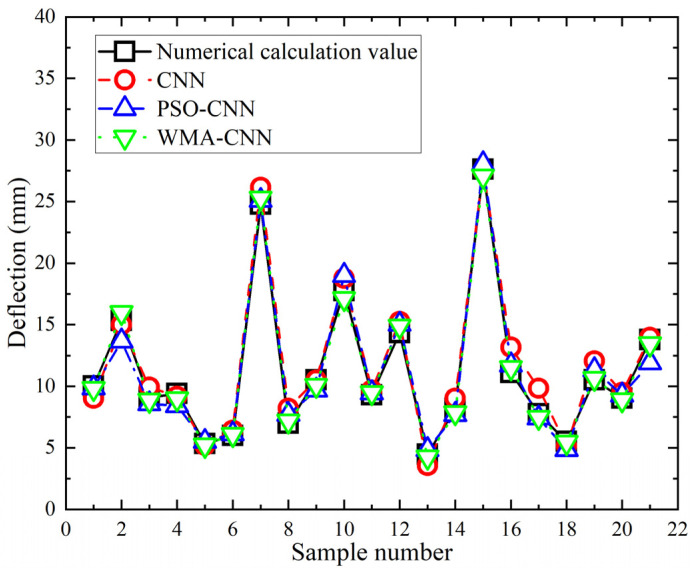
Deflection comparison of numerical values and predicted values.

**Figure 21:**
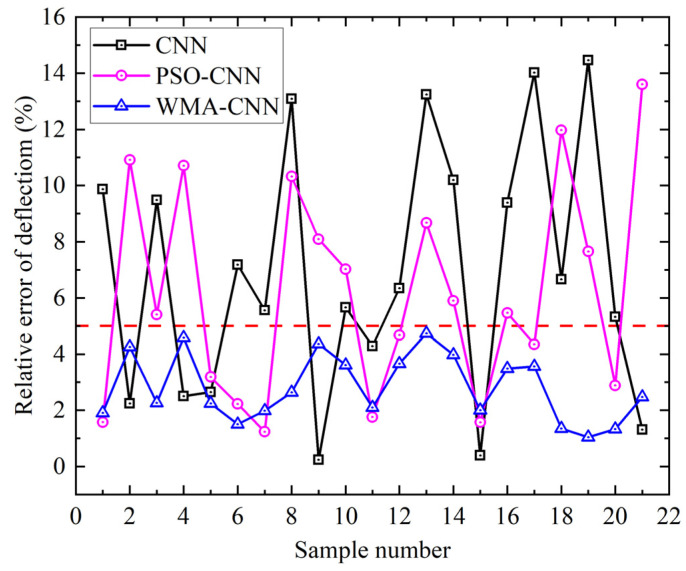
Relative error comparison of deflection.

Comparison of the predicted and numerical calculation values of twenty-one sets of deflection samples for the PSO-CNN model, the WMA-CNN model, and the CNN model is shown in Figure 20. Marked deviations are observed in CNN predictions for samples 16,17, and 19. In PSO-CNN, marked deviations are shown in samples 2 and 21. Predictions from the WMA-CNN model are closer to numerically calculated deflection values. The relative error of the predicted and numerically calculated values of twenty-one sets of deflection samples for the PSO-CNN model, the WMA-CNN model and the CNN model are shown in Figure 21. It can be seen that the maximum relative error is 14.46% for CNN, reduced to 13.60% for PSO-CNN, and further decreased to 4.73% for WMA-CNN. CNN predictions exceed 5% relative error in 14 samples, PSO-CNN in 12 samples, while all WMA-CNN values remain below 5%. The results show that the WMA-CNN model achieves better deflection prediction accuracy than both the CNN model and PSO-CNN model.

### 4.2. Analysis of Evaluation Indices for the Prediction Model

Beyond the mentioned MSE, evaluation indices for the prediction model include mean absolute error (MAE), root mean squared error (RMSE), mean absolute percentage error (MAPE), and coefficient of determination (R^2^), with their computational equations presented as follows:

(11) $$\mathrm{MAE}=\frac{1}{m}\sum_{i=1}^{m}\left|y_{i}-{\overset{⌢}{y}}_{i}\right|$$

(12) $$\mathrm{RMSE}=\sqrt{\frac{1}{m}{\sum_{i=1}^{m}\left(y_{i}-{\overset{⌢}{y}}_{i}\right)}^{2}}$$

(13) $$\mathrm{MAPE}=\frac{1}{m}\sum_{i=1}^{m}\left|\frac{y_{i}-{\overset{⌢}{y}}_{i}}{y_{i}}\right|$$

(14) $$R^{2}=1-\frac{\sum_{i=1}^{m}\left(y_{i}-{\overset{⌢}{y}}_{i}\right)}{\sum_{i=1}^{m}\left(y_{i}-{\bar{y}}_{i}\right)}$$

where $y_{i}$ represents numerical calculation values; ${\overset{⌢}{y}}_{i}$ represents predicted values; m represents the number of samples in the training set; ${\bar{y}}_{i}$ represents the mean value of numerical calculation values.

**Figure 22:**
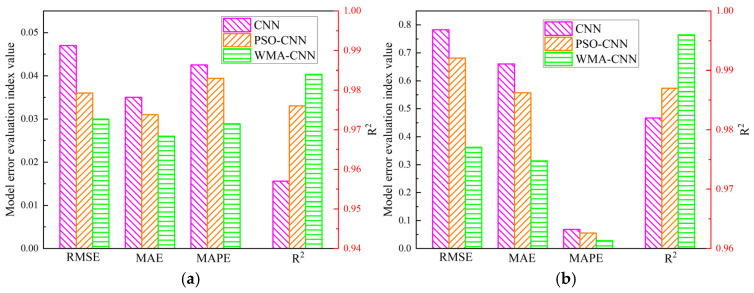
Prediction evaluation indices of the three models: (**a**) shrinkage; (**b**) deflection.

Prediction evaluation indices for CNN, PSO-CNN, and WMA-CNN are summarized in Table 7, with comparative results illustrated in Figure 22. Among the three models, the WMA-CNN model has the highest prediction accuracy. For shrinkage prediction, the coefficient of determination was the largest (R^2^ = 0.984) and the error was the smallest (RMSE = 0.030, MAE = 0.026, MAPE = 2.89%). For deflection prediction, the coefficient of determination was the largest (R^2^ = 0.996) and the error was the smallest (RMSE = 0.362, MAE = 0.314, MAPE = 2.81%). Compared to CNN model, for shrinkage prediction, the MAPE of PSO-CNN model and WMA-CNN model were reduced by 7.3% and 32.0%. For deflection prediction, the MAPE of the PSO-CNN model versus the WMA-CNN model was reduced by 19.1% and 59.0%. Therefore, for curved plate deflection prediction, the WMA-CNN model outperforms the CNN model and PSO-CNN model.

### 4.3. Validation Cases for the WMA-CNN Prediction Model

To examine the accuracy of the WMA-CNN prediction model, five cases of curved plate forming are designed for validation. The numerical calculation results of shrinkage deflection are obtained by numerical calculation. The absolute error and relative error between the prediction results and numerical calculation results of WMA-CNN model are calculated. The validation results of the algorithms are shown in Table 8. From the table, it can be seen that the relative error of shrinkage and deflection of the five cases are within 5%, which is within the acceptable range, proving that the WMA-CNN model can be used for intelligent prediction of curved plate forming.

## 5. Conclusions

In this paper, a finite element simulation model of line heating of curved plate was established based on the SDB. Its feasibility was verified by comparing finite element numerical results with experimental data. Multiple sets of sample data were obtained through Latin hypercube sampling. The deformation prediction model based on CNN was established. The relative error between CNN predictions and numerically calculated values was analyzed. Particle swarm optimization and whale migrating algorithm were introduced to optimize the weights of CNN model. Therefore, PSO-CNN model and WMA-CNN model were established. The RMSE, MAPE, MSE, and R^2^ of CNN model, PSO-CNN model, and WMA-CNN model were analyzed. Through calculation cases, the WMA-CNN model for curved plate forming was validated.

1. Numerical calculations from the TEP-FEM and SDB showed closely matched temperature and deformation results. This confirms the applicability of the SDB for line heating forming numerical calculation.
2. To realize the fast prediction of curved plate forming, a prediction model of curved plate forming deformation based on CNN was established. The CNN model is optimized by PSO algorithm and WMA algorithm, and the curved plate deformation prediction models by PSO-CNN and WMA-CNN were established. When the WMA-CNN model predicts deformation, the relative error of the sample data is within 5%. It indicates that the prediction accuracy of WMA-CNN model is better than CNN model and PSO-CNN model.
3. Among the three models, the WMA-CNN model has the highest prediction accuracy. For shrinkage prediction, the coefficient of determination was the largest (R^2^ = 0.984). For deflection prediction, the largest coefficient of determination was (R^2^ = 0.996). Compared to CNN model, for shrinkage prediction, the MAPE of PSO-CNN model and WMA-CNN model were reduced by 7.3% and 32.0%. For deflection prediction, the MAPE of the PSO-CNN model versus the WMA-CNN model was reduced by 19.1% and 59.0%. Therefore, for curved plate deflection prediction, the WMA-CNN model outperforms the CNN model and PSO-CNN model.
4. The WMA-CNN model is applied for the validation of five calculation cases. The relative error between the prediction results and the numerical calculation results is within 5%, which is within the acceptable range. It is demonstrated that the WMA-CNN model is suitable for intelligent prediction of curved plate line heating forming. This research provides reference for automated forming of hull curved plate. In the subsequent work, the research findings will be applied to predict the forming parameters of curved plates. Concurrently, the development of automated curved plate forming equipment and a real-time forming control system will be carried out. Finally, the accuracy of the established parameter prediction system will be validated through dedicated curved plate forming experiments, thereby laying a foundation for the practical implementation of intelligent line heating technology.

## Figures and Tables

**Table 1:** Heat affected zone temperature distribution.

Position	Temperature 20 mm from the Center (°C)	Temperature 10 mm from the Center (°C)	Center Position Temperature (°C)
First layer	520.3	718.6	808.5
Second layer	/	613.1	680.9
Third layer	/	567.2	618.4
Fourth layer	/	/	583.1
Fifth layer	/	/	/

**Table 2:** Parameters of finite element model.

Parameter	Value	Unit
Plate length	3	m
Plate width	1.5	m
Plate thickness	14	mm
Curvature radius	5	m
Heating line length	300	mm

**Table 3:** Different mesh size schemes.

Mesh Size of Dense Area (mm)	Mesh Size of Transition Area (mm)	Mesh Size of Sparse Area (mm)
5	10	25
8	16	40
10	20	50
12	24	60
16	32	80
18	36	90
20	40	100
25	50	125
28	56	140

**Table 4:** Experimental and numerical calculation results of shrinkage.

Distance to the Initial Point of Heating Line (mm)	0	75	150	225	300
Experimental data (mm)	0.27	0.49	0.75	1.07	1.35
Numerical calculation (mm)	0.269	0.509	0.741	1.078	1.391
Absolute error (mm)	0.001	0.019	0.009	0.008	0.041
Relative error (%)	0.37	3.88	1.20	0.75	3.03

**Table 5:** Sampling results.

No.	Plate Length (m)	Plate Width (m)	Curvature Radius (m)	Plate Thickness (mm)	Heating Line Length (m)	Shrinkage (mm)	Deflection (mm)
1	3.2	1.2	4	12	0.3	1.18	17.76
2	4.8	1.6	4.3	8	0.3	1.31	30.88
3	3.7	1.6	3	24	0.3	0.91	8.65
4	3.6	2.3	2.8	24	0.3	0.88	8.02
5	4.1	1.5	2.3	24	0.2	0.65	11.12
⋯	⋯	⋯	⋯	⋯	⋯	⋯	⋯
100	4	1.9	3.7	24	0.2	0.65	13.50
101	4.3	1.7	1.9	8	0.4	1.19	13.78
102	5	2.6	4.4	24	0.2	0.67	13.86

**Table 6:** Normalized sample data.

No.	Plate Length	Plate Width	Curvature Radius	Plate Thickness	Heating Line Length	Shrinkage	Deflection
1	0.400	0.125	0.706	0.200	0.500	0.833	0.332
2	0.933	0.375	0.794	0.000	0.500	0.988	0.660
3	0.567	0.375	0.412	0.800	0.500	0.512	0.104
4	0.533	0.813	0.353	0.800	0.500	0.476	0.089
5	0.700	0.313	0.206	0.800	0.000	0.202	0.166
⋯	⋯	⋯	⋯	⋯	⋯	⋯	⋯
100	0.667	0.563	0.618	0.800	0.000	0.202	0.226
101	0.767	0.438	0.088	0.000	1.000	0.845	0.233
102	1.000	1.000	0.824	0.800	0.000	0.226	0.235

**Table 7:** Prediction evaluation indices.

Variables	Model	MSE (mm^2^)	RMSE (mm)	MAE (mm)	MAPE	R^2^
Shrinkage	CNN	0.0020	0.045	0.034	0.0425	0.963
	PSO-CNN	0.0013	0.036	0.031	0.0394	0.976
	WMA-CNN	0.0009	0.030	0.026	0.0289	0.984
Deflection	CNN	0.6133	0.783	0.660	0.0686	0.982
	PSO-CNN	0.4633	0.681	0.557	0.0555	0.987
	WMA-CNN	0.1312	0.362	0.314	0.0281	0.996

**Table 8:** Cases validation for WMA-CNN.

Parameters and Indices	Case 1	Case 2	Case 3	Case 4	Case 5
Plate length (m)	4.4	4.6	4	4.7	3.6
Plate width (m)	2	1.9	1.7	2.5	1.7
Plate thickness (mm)	28	8	16	20	20
Curvature radius (m)	3	2.1	3.5	4.3	3.4
Heating line length (m)	0.4	0.4	0.2	0.2	0.3
Calculated shrinkage (mm)	0.51	1.20	0.77	0.71	0.9
Predicted shrinkage (mm)	0.529	1.243	0.758	0.736	0.861
Calculated deflection (mm)	9.21	15.18	17.48	15.52	11.32
Predicted deflection (mm)	9.463	14.589	18.250	14.936	11.110
Absolute error of shrinkage (mm)	−0.019	−0.043	0.012	−0.026	0.039
Relative error of shrinkage	3.73%	3.58%	1.56%	3.66%	4.33%
Absolute error of deflection (mm)	−0.253	0.591	−0.770	0.584	0.210
Relative error of deflection	2.75%	3.89%	4.41%	3.76%	1.86%

## Data Availability

The original contributions presented in this study are included in the article. Further inquiries can be directed to the corresponding author.
